# Decision-Making for Surgery in the Management of Patients with Univentricular Heart

**DOI:** 10.3389/fped.2015.00061

**Published:** 2015-07-27

**Authors:** Ryan Robert Davies, Christian Pizarro

**Affiliations:** ^1^Nemours Cardiac Center, A. I. duPont Hospital for Children, Wilmington, DE, USA; ^2^Thomas Jefferson University, Philadelphia, PA, USA

**Keywords:** univentricular heart, congenital heart disease, palliation, single ventricle, aortopulmonary shunt, Blalock–Taussig procedure, Fontan procedure, hemi-Fontan procedure

## Abstract

A series of technical refinements over the past 30 years, in combination with advances in perioperative management, have resulted in dramatic improvements in the survival of patients with univentricular heart. While the goal of single-ventricle palliation remains unchanged – normalization of the pressure and volume loads on the systemic ventricle, the strategies to achieve that goal have become more diverse. Optimal palliation relies on a thorough understanding of the changing physiology over the first years of life and the risks and consequences of each palliative strategy. This review describes how to optimize surgical decision-making in univentricular patients based on a current understanding of anatomy, physiology, and surgical palliation.

## Introduction

Over the past 30 years, there has been a dramatic improvement in the survival of patients with univentricular heart (1, 2). The development of innovative techniques for surgical palliation – including the Fontan (3) and Norwood procedures (4) – has been followed by surgical refinement and advances in perioperative management. The list of technical refinements is long, including: the use of staged palliation, branch pulmonary artery banding, the hybrid procedure, comprehensive second stage palliation, and various technical modifications to both the Norwood procedure and the superior and total cavopulmonary connections (TCPC).

The ultimate goal of staged univentricular palliation is to normalize the volume and pressure work of the functional ventricle while pumping blood fully saturated with oxygen, regardless of the underlying cardiac anatomy (5). However, the elevated pulmonary vascular resistance (PVR) present in the early post-natal period means that attainment of this long-term goal must be delayed, resulting in the need for a staged management strategy in most cases. Accordingly, post-natal palliation requires that the pulmonary and systemic circulations remain in parallel, while pulmonary blood flow is controlled, allowing for the proper development and maturation of the pulmonary vascular bed. Subsequently, the conversion to a cavopulmonary connection allows the transition to a circulatory arrangement with the circulations connected in series. In this context, appropriate decision-making relies on a thorough understanding of the anatomy and physiology of the univentricular heart at each stage of palliation, as well as a comprehensive knowledge of the advantages and disadvantages of the various management strategies.

## Perinatal Management

Early perinatal management of patients with univentricular heart is focused on identification of the anatomy (which may constitute an immediate risk to the patient after birth) and on stabilization with the usual measures to control the volume and pressure load to the ventricle while enabling adequate systemic delivery of oxygen. These goals must be achieved regardless of the underlying anatomic substrate (whether hypoplastic left heart syndrome, an unbalanced atrioventricular septal defect, or tricuspid atresia). Attention should be paid to the delicate and dynamic balance between the systemic and PVR ratio, leading to ongoing re-evaluation and adjustments in management as the PVR falls (6).

Evaluation of the neonate with single ventricle is directed at identifying answers to the following questions: (1) is there a reliable source of systemic blood flow? (2) is there a reliable source of pulmonary blood flow? (3) Is there any impediment to pulmonary venous return? and (4) Is there an appropriate balance between the systemic and pulmonary circulations? (7). Non-invasive echocardiography is most commonly able to provide these answers; cardiac catheterization is rarely required.

### Ductus arteriosus

In cases when there is a significant impediment to pulmonary or systemic circulations, the ductus arteriosus can be used to provide a reliable source of pulmonary or systemic blood flow. Patients in whom a patent ductus arteriosus is necessary may be divided into those with ductal-dependent *pulmonary* circulation (e.g., pulmonary atresia with intact ventricular septum, or tricuspid atresia) and those with ductal-dependent *systemic* circulation (e.g., hypoplastic left heart syndrome). In either case, maintenance of ductal patency is critical to providing adequate systemic oxygen delivery.

Since, initially described by Olley and colleagues in 1976 and later by the Green Lane Unit (8, 9), prostaglandin E (PGE 1) remains the mainstay of medical treatment to maintain patency of the ductus arteriosus. While treatment with PGE 1 has transformed the early management of neonates with ductal-dependent lesions, it is not without side effects (10). Particularly concerning among single-ventricle patients are apnea and the potential for compromise of gastrointestinal perfusion (11, 12). These side effects are dose-dependent; therefore, the dose should be titrated to the lowest level required to maintain ductal patency (11). In fact, in nearly all patients, a dose of 0.01 mcg/kg/min should be adequate for continued ductal patency while limiting the risk of significant side effects (11).

### Balancing systemic and pulmonary blood flow

In cases where the pulmonary and systemic circulations are connected in parallel, blood leaving the functional ventricle may enter either the pulmonary artery or the systemic circulation. The underlying anatomy, and in particular, the presence of either pulmonary or systemic outflow obstruction will have an important influence on the relative balance between pulmonary and systemic blood flow (Table 1). In the absence of obstruction, the relative blood flow to each circulatory component depends predominantly on relative balance between pulmonary and systemic vascular resistances. This is particularly important when the ductus arteriosus remains patent. In this scenario, there is potential for continuous diastolic runoff away from the systemic and into the pulmonary circulation, making this balance even more challenging, with the potential for dramatic effects on hemodynamic stability and systemic oxygen delivery. In cases with significant right or left outflow obstruction, an earlier surgical palliation may be required when non-surgical manipulations are inadequate to overcome the resulting imbalance in circulation or to prevent the need for a prolonged administration of PGE1 and the potential adverse effects and consequences associated with it.

**Table 1:** **Categorization of pulmonary and systemic outflow obstruction and its consequences in the univentricular heart**.

Systemic outflow	Pulmonary outflow	Examples	Ventricular volume load	Pulmonary blood flow	Consequences
Unobstructed	Unobstructed	Unbalanced atrioventricular septal defect	↑↑↑	↑	Congestive heart failure due to pulmonary overcirculation as pulmonary vascular resistance falls
Obstructed	Unobstructed	Hypoplastic left heart syndrome	↑↑	↑↑↑	Severe congestive heart failure due to pulmonary overcirculation as the pulmonary vascular resistance falls. inadequate systemic cardiac output
Unobstructed	Obstructed	Tricuspid atresia with pulmonary atresia	↑	↓↓	Progressive and severe cyanosis due to inadequate pulmonary blood flow

*Adapted from Walker et al. (7)*.

Balancing the distribution of cardiac output is especially important in those patients with a circulation connected in parallel at the arterial level, which commonly leads to excessive pulmonary blood flow and pulmonary over circulation. This is even more critical in the presence of ventricular dysfunction, atrioventricular valve regurgitation, or rhythm disturbance. Through manipulations in inspired respiratory gases, the balance between systemic and pulmonary circulations can be modified. Oxygen is a pulmonary vasodilator, while hypercarbia is a potent pulmonary vasoconstrictor, and also a cerebral vasodilator (13) Respiratory gas mixtures low in oxygen and those with added carbon dioxide have been used to increase PVR and decrease the Qp/Qs. In fact, the addition of carbon dioxide to the inspired gas mixture increases PVR, decreases the volume load on the ventricle, and improves systemic cardiac output (13, 14). Similar vasoconstriction has been observed using sub-atmospheric inhaled oxygen concentrations to induce alveolar hypoxia; however, this method lacks the cerebral vasodilatory effect (13). In some cases, mechanical ventilation and paralysis may be required in order to provide precise control over pCO_2_ and pO_2_; but the manipulation of respiratory gas exchange is often sufficient to provide balanced pulmonary blood flow in the early post-natal period (13).

### Challenges in early management

Patients presenting with ventricular dysfunction, obstructed pulmonary venous return, significant atrioventricular valve regurgitation, or non-cardiac disease (e.g., sepsis or gastrointestinal anomalies) present important challenges. The presence of atrioventricular valve regurgitation results in an additional volume load on the already loaded ventricle. This commonly leads to congestive heart failure and not infrequently a gradual and progressive onset of ventricular dysfunction. Early control of pulmonary blood flow to reduce additional volume loading is essential. Not infrequently – particularly among patients with a single right ventricle or those with a common atrioventricular valve – control of pulmonary blood flow can be associated with a significant reduction of the valve regurgitation (15, 16). In this scenario, an intervention should be performed without delay.

The presence of ventricular dysfunction can be associated with late diagnosis or intrinsic myocardial dysfunction. Commonly, the dysfunctional ventricle is unable to meet the work requirements of the single-ventricle circulation, leading to circulatory failure and end organ dysfunction. As stated previously, early attention to reducing the volume and pressure load on the ventricle is critical. If obstruction to systemic blood flow is present, an unobstructed pathway must be secured with the use of PGE1 or with an intervention. While manipulation of respiratory gas exchange in combination with inotropic support may be sufficient, some patients will require early surgical control of pulmonary blood flow. Depending on the underlying anatomy, this may be performed using either a main pulmonary artery band or, if patency of the ductus arteriosus is necessary, branch pulmonary artery banding (see below). In all patients, this will reduce the Qp/Qs, volume load, ventricular work, and usually will lower the end-diastolic pressure over time. Moreover, in those with ductal-dependent circulations (such as HLHS), it may have the added benefit of augmenting systemic diastolic pressure and enhancing coronary perfusion.

In contradistinction, obstructed pulmonary venous return is often a serious and frequently insurmountable problem. The timing of intervention varies, from emergent in the case of intact atrial septum to semi-elective in the case of moderate obstruction or less. Alternatively, if the anomalous pulmonary venous drainage is unobstructed, control of pulmonary blood flow may reduce the pulmonary venous gradient. Repair can then be deferred to a later date – ideally the second-stage procedure (17).

Although an atrial septectomy or repair of total anomalous pulmonary venous connections may resolve an anatomic obstruction (18), fetal development of the pulmonary vasculature in the setting of obstructed pulmonary venous drainage is associated with irreversible alterations in pulmonary vascular structure (19). This results in elevated PVR, which is associated with significant morbidity and mortality following cavopulmonary connection. Thus, overall outcomes among patients with single ventricle and obstructed pulmonary venous return are poor, especially in the setting of heterotaxy syndrome (18, 20–22). In the recent years, although novel attempts to relieve pulmonary venous obstruction during fetal life have been associated with improved early survival, this has commonly been associated with important morbidity and mortality during follow up (18).

Finally, the management of single-ventricle patients with non-cardiac congenital anomalies or with postnatal illness (particularly sepsis) remains difficult. In this scenario, caution should be utilized when choosing conventional treatment measures. While systemic vasoconstrictors may be essential for maintaining blood pressure in septic patients, in univentricular heart patients, they can lead to disproportionate increases in SVR resulting in the rapid development of pulmonary overcirculation, volume overload, and poor oxygen delivery. In hypotensive patients, clinical and ecocardiographic evaluation should assist in identifying the underlying mechanism: vasodilatation, poor cardiac function, or maldistribution of cardiac output. In the case of sepsis, epinephrine provides the most effective support. The use of dopamine in single-ventricle patients is controversial; as it has been shown to increase the systemic oxygen demand offsetting any improvement in systemic oxygen delivery (23). Pure vasoconstriction agents have a significant risk of increasing the systemic more than PVR, resulting in systemic hypoperfusion and cardiac volume overload (1). In addition, the presence of a systemic inflammatory response and poor oxygen diffusion in the lungs may suggest the need for supplemental oxygen; however, this can result in pulmonary vasodilation and heart failure. These patients will commonly require early surgical palliation to control pulmonary blood flow as well as invasive monitoring in order to allow for optimization of the circulatory physiology and treatments directed at the non-cardiac pathology.

## Initial Surgical Palliation

The goals of initial palliation are to provide unobstructed systemic blood flow, well-balanced pulmonary and systemic circulations with controlled pulmonary blood flow, and unobstructed pulmonary and systemic venous return (including unrestricted atrial level mixing of venous returns). Although the long-term goals include normalization of the ventricular volume load and provision of normal systemic oxygen delivery, the elevated PVR characteristic of the newborn period requires a staged approach to achieve these long-term goals. As part of the initial surgical palliation, relief of any systemic outflow tract and pulmonary venous obstructions must also be undertaken. Thus, the precise procedure to accomplish the goals will depend on the underlying anatomy but may include the creation of a reliable and controlled source of pulmonary blood flood through a systemic–pulmonary shunt, limitation of pulmonary blood flow via main pulmonary banding, repair of aortic coarctation and aortic arch hypoplasia, or a combination of these (including the Norwood procedure). Relief of any pulmonary venous obstruction by either reconstruction of stenotic anomalous pulmonary venous connections or a complete atrial septectomy should be performed as needed.

### Timing of initial intervention

The timing of the initial intervention directed at regulating the pulmonary blood flow (Qp) is determined by the severity of the baseline flow imbalance. In patients with reduced pulmonary blood flow and low Qp, the degree of cyanosis is the best indication for when to proceed with surgical palliation. Creation of a reliable and effective source of pulmonary blood flow with a shunt procedure (see below) should be undertaken when severe cyanosis (O_2_Sat <70%) is present (24). As part of the preoperative evaluation, other sources of cyanosis, including reversible lung disease, anemia, and obstruction to pulmonary venous flow need to be ruled-out (24).

In contradistinction, in patients with excessive pulmonary blood flow, the onset of signs and symptoms consistent with volume overload and occasionally congestive heart failure (growth failure, tachycardia, tachypnea, the need for mechanical ventilation) suggest the need for pulmonary banding. Signs will usually worsen as the PVR falls postnatally. Occasionally, there is a reasonable balance between systemic and pulmonary circulation, while the pulmonary vascular bed is protected from high pressure and high flow. In some cases, these patients can exhibit growth and development in the first months of life, while the pulmonary vascular bed matures and develops. In this scenario, patients may then proceed directly to a superior cavopulmonary connection at an appropriate age.

### Control of pulmonary blood flow–pulmonary artery banding

In patients with excessive Qp and signs of heart failure, control of pulmonary blood flow is essential to permit somatic growth and eliminate the volume load to the ventricle while the normal post-natal decrease in PVR takes place (6, 25). Most commonly, this is performed using a band on the main pulmonary artery, although patients with ductal-dependent systemic blood flow may undergo a hybrid procedure involving branch pulmonary artery band placement (see below).

It should be noted, that in patients with single-ventricle and discordant ventriculo arterial connection, pulmonary artery banding can lead to significant systemic outflow obstruction. In these patients, systemic outflow is dependent on flow through the ventricular septal defect (bulboventricular foramen) and the hypoplastic outflow chamber. In this scenario, pulmonary artery banding is usually associated with significant myocardial hypertrophy, leading to outflow obstruction in as many as 70–100% of patients (26, 27). Moreover, if surgical reconstruction is necessary to alleviate this issue, the risk is particularly high (27–30). Based on this experience, a Damus–Kaye–Stansel (DKS) or a modified Norwood procedure (with pulmonary blood flow provided by a systemic-to-pulmonary shunt) has been shown to provide a reliable systemic outflow, and avoids the need for subsequent reinterventions (31). Nevertheless, other reports still suggest acceptable outcomes with a strategy of arch reconstruction and pulmonary artery banding (29, 32–34). If this strategy is chosen, close surveillance to evaluate early development of restriction is mandatory, and a low-threshold for DKS anastomosis either before or at the superior cavopulmonary connection is critical. In the long-run, failure to recognize subaortic obstruction and the consequent ventricular hypertrophy may result in compromised Fontan candidacy (35, 36).

### Augmentation of pulmonary blood flow

Among patients with inadequate pulmonary blood flow (either preoperatively or as the result of initial palliation as in the Norwood procedure), augmentation of pulmonary blood flow through the use of a systemic-to-pulmonary shunt may be necessary. Multiple techniques are of primarily historical importance, including the classic Glenn, the classic Blalock–Taussig, the Waterston, and the Potts shunts. In current practice, nearly all shunts are a modified Blalock–Taussig shunt (mBTS) consisting of a PTFE graft connecting the proximal innominate artery and the right pulmonary artery.

In most cases, the shunt is performed via a median sternotomy with the variable use of cardiopulmonary bypass. This incision has several advantages over the traditional lateral thoracotomy, including the ability to ventilate both lungs, more central placement on the pulmonary arteries (minimizing the risk of lobar branch compromise), access for ligation of the arterial duct, and the ease of cannulation for cardiopulmonary bypass where required (24). In addition, avoidance of a thoracotomy mitigates the development of systemic-to-pulmonary artery collaterals and prevents morbidity related to the lung parenchyma or the pleural space, which could have a deleterious effect on Fontan candidacy. In general, post-procedural arterial oxygen saturation of 75–85% indicates an appropriate sized shunt with a Qp:Qs approaching 1:1.

**Figure 1:**
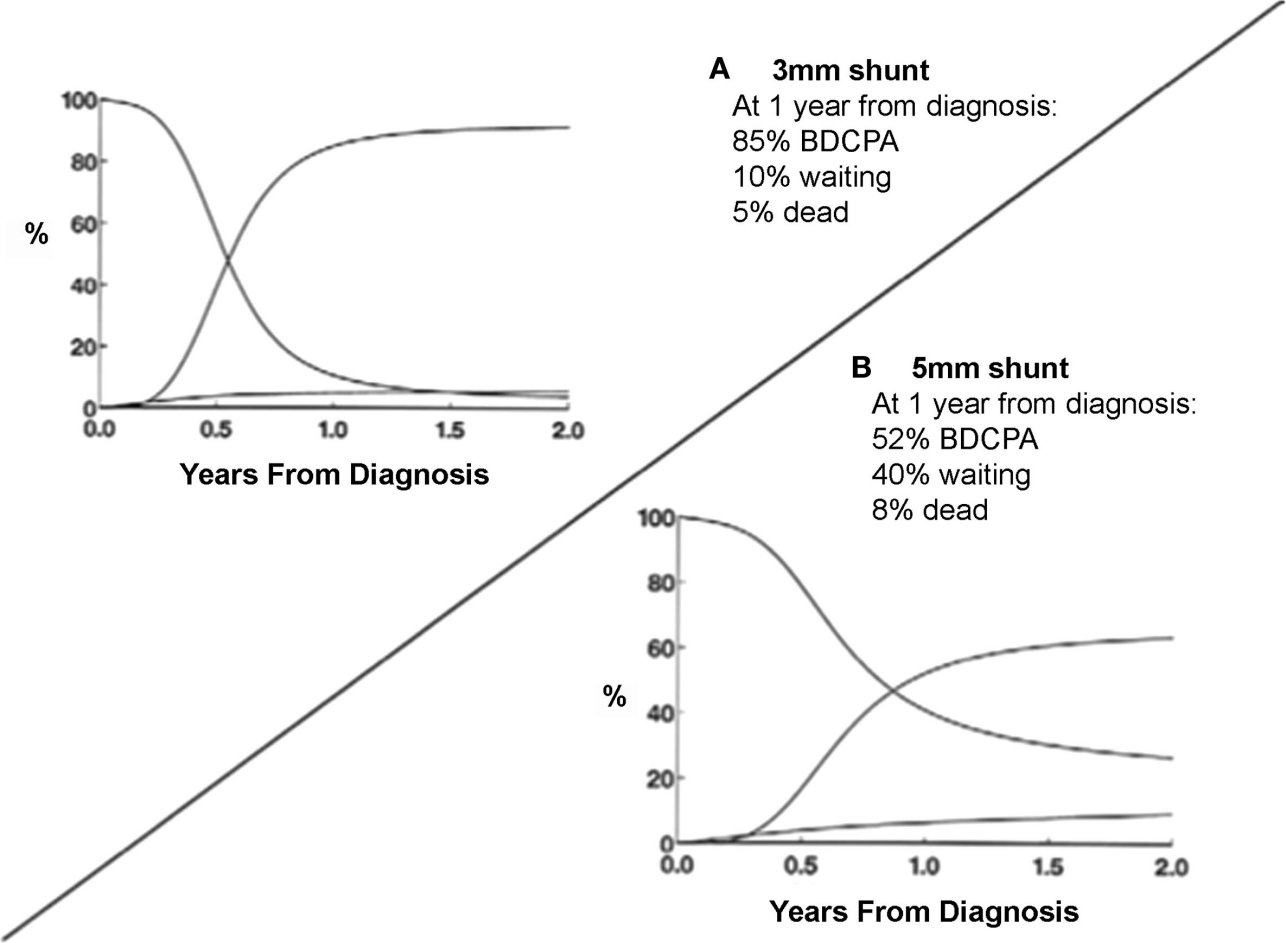
**Smaller systemic-pulmonary arterial shunt size resulted in decreased mortality and increased transition rates to BDCPA**. **(A)** Use of 3-mm shunt resulted in 85% of patients having BDCPA by 1 year, 10% still in palliated state, and only 5% of dead. **(B)** Use of larger 5-mm shunt resulted in much slower transition rate and slightly increased rate of death without BDCPA [from Karamlou et al. (37)].

The diameter of the PTFE shunt is the primary determinant of its resistance. Alterations in the length of the tube or in its position on the arterial tree play a smaller, but still important role. In neonates weighing 3.5 kg, a 3.5-mm shunt is usually appropriate. In slightly larger infants or those with smaller pulmonary arteries, placing the shunt more proximally within the systemic circulation may provide additional flow without the dramatic decrease in resistance apparent with a step-up to a 4-mm shunt. However, shunts originating in locations other than the innominate artery may have a higher early mortality risk, although confounding with morphologic variation preventing the use of the innominate artery may play an important role (37). Despite the popularity of the 3.5-mm standard, there is growing evidence that the use of smaller shunts may improve outcomes – both for patients with hypoplastic left heart syndrome and tricuspid atresia (Figure 1) (37, 38). However, this may be associated with a higher incidence of thrombosis and need for reintervention (39). Unfortunately, there is no mathematical formula to define the optimal shunt size in a specific patient. Several factors will determine pulmonary blood flow including the site of insertion and the diameter of the shunt, PVR, and the technical skill and experience of the surgeon in obtaining a 3.5-mm opening using a 3.5-mm graft (24).

The use of aspirin following the placement of a systemic-to-pulmonary shunt (especially with PTFE) is a common practice but based primarily on observational data. A retrospective observational study by Li and colleagues suggests a beneficial impact of aspirin in preventing shunt thrombosis and improving survival in shunted patients (40). More recently, a randomized, controlled trial failed to show a benefit of clopidogrel over placebo in transplant-free survival, but nearly 90% of patients in the trial were on aspirin, suggesting that the message is really that clopidogrel and aspirin is no better than aspirin (41). In fact, *post hoc* analysis of that population suggested a benefit with the use of aspirin in this population, with a 40% relative risk reduction in the incidence of the primary outcomes (death, transplantation, or shunt thrombosis) (41). Based on our own experience, aspirin does not appear important in preventing shunt thrombosis (personal communication). Thrombotic complications are common in these patients, and may influence mortality even in the presence of a patent systemic-to-pulmonary shunt (42). A randomized trial would be valuable in confirming the utility of aspirin in preventing these complications.

Preservation of antegrade pulmonary flow may lead to pulmonary overcirculation and volume overloading. This can be particularly deleterious among patients with atrioventricular valve regurgitation, in whom maintaining pulmonary arterial patency is associated with worsening regurgitation and higher risk of early mortality (37). Not infrequently, antegrade pulmonary blood flow is preserved based on the notion that it can improve survival in case of shunt thrombosis; however, this has never been demonstrated. More importantly, due to the presence of competitive blood flow, the presence of antegrade pulmonary blood flow has been associated with trends toward increased shunt thrombosis and mortality in multiple studies (43, 44).

Although rare in univentricular patients, the presence of a dynamic component to pulmonary flow limitation increases the complexity of early postnatal management. In the patient with a single functional ventricle, hyper cyanotic spells may result in a rapid deterioration of ventricular function, myocardial ischemia, and death. However, preservation of the native pulmonary blood flow at the time of insertion of a systemic-to-pulmonary artery shunt in order to provide adequate Qp during episodes of dynamic obstruction may lead to a extremely challenging management of excessive Qp at baseline. In these cases, it seems preferable to ligate and divide the main pulmonary artery, relying instead on a single and fixed source of pulmonary blood flow.

### Obstruction to systemic outflow

Provision of a reliable and unobstructed systemic outflow pathway represents one of the main tenets in the management of single-ventricle patients. Failure to relieve systemic outflow obstruction during initial palliation has several deleterious consequences. If the outflow obstruction is distal to the source of pulmonary blood flow (subaortic obstruction in a patient with a PA band, or a coarctation in a patient with an mBTS, aortic stenosis in a patient with an RV–PA conduit), the result will be increased pulmonary blood flow and lower systemic output. This leads to pulmonary overcirculation, inadequate protection of the pulmonary vascular bed associated with the elevation of PVR, and ultimately increased volume load on the single ventricle. Most importantly, the outflow obstruction results in an important pressure load on the ventricle with consequent hypertrophy, increased myocardial stiffness, and elevation in end-diastolic pressure. At the time of conversion to a cavopulmonary connection (and the loss of the systemic blood pressure as a driving force for pulmonary blood flow), elevations in end-diastolic pressure increased PVR and may preclude Fontan completion or result in early Fontan failure and poor survival (36, 45–47).

The most common procedures used in the relief of systemic outflow tract obstruction are the DKS and Norwood procedures. In both cases, the main pulmonary artery is used as the main pathway for the systemic outflow. In cases when the systemic outflow obstruction is associated with aortic arch hypoplasia and/or coarctation of the aorta, then Norwood procedure is the procedure of choice (4).

As noted above, the threshold for performing either a DKS or a Norwood procedure in patients with a univentricular heart and potential or mild systemic outflow obstruction is a matter of controversy. If creation of a DKS is considered, placement of a pulmonary band should be tempered by the distortion of the pulmonary (neo-aortic) root and valve, which can be associated with neo-aortic insufficiency at the time of DKS. The negative feedback loop initiated by the presence of ventricular outflow obstruction stimulates myocardial hypertrophy leading to worsening outflow obstruction and the potential for long-term myocardial alterations as the result of early pressure loading of the ventricle. Given these considerations, the threshold for performance of a procedure to relieve outflow obstruction should be low with the goal to provide a durable and reliable solution that would avoid the need for repeated reinterventions. Evidence-based guidelines would require a prospective trial.

#### Stage 1 Norwood Procedure

The Norwood procedure is commonly performed among patients with univentricular hearts and systemic outflow obstruction. In spite of nearly three decades of experience, little consensus exists regarding the most appropriate source of pulmonary blood flow, techniques of intraoperative management, and cerebral protection, and whether hybrid or conventional procedures provide the most appropriate palliation.

##### Choice of shunt

Since initially described, the source of pulmonary blood flow has evolved over time, including a direct RV to PA conduit, central shunt, and modified BT shunt (48–50). The RV–PA conduit has the advantage of eliminating the systemic diastolic run-off into the pulmonary circulation that causes reversal of flow in the ascending aorta and coronary arteries among patients palliated with an mBTS (51). This flow reversal places patients at-risk for coronary ischemia and has been theorized to contribute to interstage mortality (52, 53). However, concern still exists about the impact of the ventriculotomy on late ventricular dysfunction and its use has been associated with a higher number of interventions prior to subsequent palliation (54).

**Figure 2:**
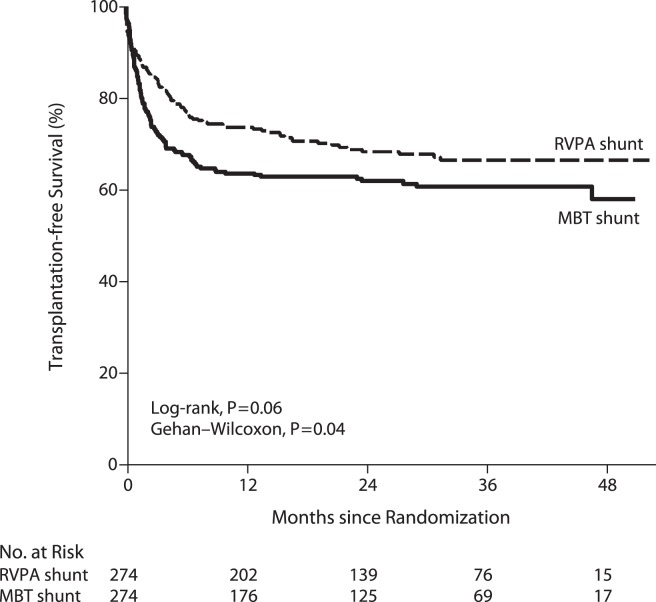
**Kaplan–Meier curves for transplantation-free survival among all infants who underwent the Norwood procedure, according to the intention-to-treat analysis**. *P* = 0.02 for the difference in the treatment effect for the period before and the period after 12 months. MBT denotes modified Blalock–Taussig, and RVPA denotes right ventricle–pulmonary artery [from Ohye et al. (53)].

**Figure 3:**
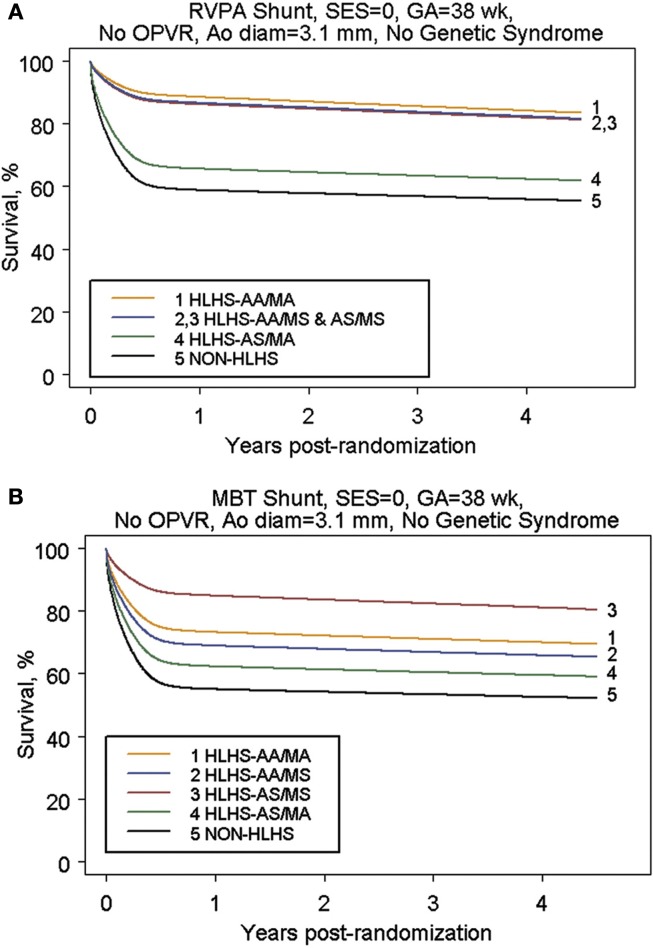
**(A)** Parametric survival curve by anatomic subtype of subjects undergoing RVPAS. **(B)** Parametric survival curve by anatomic subtype of subjects undergoing MBTS [from Tweddell et al. (20)].

In order to answer these questions, a multi-center randomized trial was undertaken to compare the two shunt types (53). Although there was a 10% reduction in mortality at 1 year with the use of a right ventricle to pulmonary artery conduit, no significant difference was observed in transplant-free survival at 2 years following Norwood procedure (Figure 2) (53). This early difference in mortality is consistent with the physiologic advantages of the RV–PA conduit: elimination of diastolic run off with a resulting less precarious circulatory balance during the early post-operative period. However, patients with an RV–PA conduit underwent more interventions on the pulmonary artery (53, 55). A more detailed analysis of this cohort revealed that the use of an RV to PA conduit offered a significant initial improvement on transplant-free survival among those patients with an atretic aortic valve who were 2.5 kg or bigger (Figure 3) (20). Beyond that, it appears that surgeon and institutional familiarity with a particular strategy (whether mBTS or RV–PA conduit) may be the most important determinant of institutional outcomes. However, it is likely that centers with less familiarity with the Norwood procedure may have more stable immediate post-operative courses and improved survival with the use of the RV–PA conduit (53).

Our current practice is to prefer the RV–PA conduit in patients with aortic atresia where the risk of coronary ischemia is particularly high in the absence of antegrade aortic blood flow. Otherwise patients receive an mBTS due to our concerns regarding the need for additional interventions and long-term impact on ventricular function. Importantly, ongoing modifications of the RV–PA conduit, including the use of ring reinforced grafts and dunking of the graft into the ventricle (56), may decrease the need for interventions and mitigate the negative aspects of this shunt. Continued evaluation of the optimal strategy is important as further technical modifications are introduced.

##### Hybrid versus traditional procedure

The combination of stenting of the ductus arteriosus and banding of the branch pulmonary arteries as initial palliation for hypoplastic left heart syndrome was initially described by Gibbs et al. in 1993 (57). It was thought that moving the complex cardiac surgical procedure out of the high-risk neonatal period would enable both improved survival and improved neurodevelopmental outcomes (58–61). Although an increasing number of hybrid procedures have been performed, an attendant improvement in outcomes, including neurodevelopmental function has not been realized (59, 60, 62–64). In spite of these results, enthusiasm for its use among high-risk patients continues and few centers have adopted the use of the hybrid for all patients (62). In theory, waiting for definitive palliation until patients are beyond the critical neonatal period might mitigate the risk associated with low birth weight, early gestational age, aortic atresia – all factors known to increase mortality following stage 1 Norwood procedures – and provide the time to address significant associated conditions (20, 65). Several centers have applied the hybrid procedure selectively to patients in these categories (63, 66–72).

However, with additional follow up, it has become apparent that the hybrid procedures can mitigate only some of the risk associated with these factors. Consistent with broad results in surgery among patients with low birth weight (73), it does not appear that the months of potential growth prior to surgical palliation result in improved outcomes. Furthermore, the placement of branch PA bands in infants <2 kg has significant technical challenges. Small changes in the diameter of the band may result in large alterations in the relative intraluminal diameter. Ideal band tightness and balanced pulmonary blood flow are difficult to achieve. Similarly, patients with aortic atresia continue to have a high-risk for mortality both in the interstage period and following the comprehensive second stage palliation (63). This may reflect the ongoing risk of myocardial ischemia as a result of coronary arterial dependence on retrograde arch flow between the hybrid procedure and the comprehensive second stage (61). Even in the absence of clinically evident preductal coarctation, ongoing subclinical ischemia may result in a myocardium less able to tolerate the long period of myocardial ischemia at the comprehensive second stage. While the use of the reverse BT shunt may mitigate some of this risk, it adds to the complexity of a procedure whose strongest argument is its simplicity (74).

**Figure 4:**
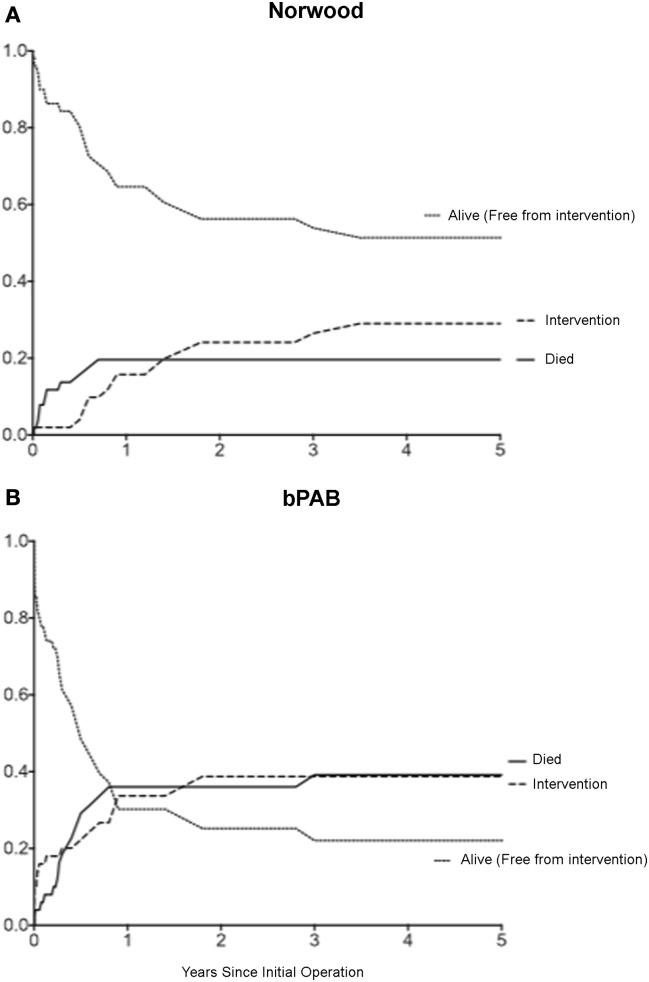
**Competing risks analysis of patients after: (A) Norwood operation; (B) removal of bilateral pulmonary arterial banding (bPAB)**. Cumulative incidence of three competing outcomes is shown: death (solid black line), intervention (dashed black line), and alive without intervention (dotted black line). Comparison of cumulative incidence between groups: death (*p* = 0.057), intervention (*p* = 0.15) [from Davies et al. (66)].

Finally, it is clear that patients undergoing hybrid palliation are affected by the obligatory distortion of the central pulmonary arteries and pulmonary arterial growth, resulting in an increased rate of pulmonary arterial interventions (Figure 4) (59, 61, 66). Although the need for pulmonary arterial interventions may be related to technical aspects of the comprehensive second stage, our data would suggest that the duration of bPAB influences the risk of intervention. Patients with branch PABs in place for longer than 90 days have higher rates of pulmonary arterial intervention (66). However, Fontan candidacy seems to be unaffected by the use of the hybrid procedure (61, 63), so it remains unclear whether these additional pulmonary arterial interventions have the feared negative long-term consequences.

Given the potential morbidity of the hybrid procedure and the inability to demonstrate a significant improvement in outcomes, its usefulness remains a matter of controversy. The use of branch PABs for the temporary control of pulmonary blood flow seems particularly advantageous in the setting of initial hemodynamic instability refractory to usual medical management, reversible risk factors, or need for an immediate non-cardiac surgical intervention (TE fistula, duodena atresia) (63, 67, 70, 75, 76). In the setting of reversible risk factors, such as, late diagnosis with end organ dysfunction infection, NEC, sepsis, etc., branch pulmonary artery bands allow for the control of pulmonary blood flow and the optimization of systemic cardiac output and coronary perfusion while allowing time for the resolution of these important co-morbidities, which are known risk factors for poor outcome. Following clinical improvement, patients can be palliated with a traditional stage 1 Norwood, also known as rapid stage I Norwood, avoiding the challenges of either long-term branch PA banding or the subsequent comprehensive second stage procedure (66, 75). Controversy still surrounds the role for hybrid palliation among centers with less experience with the traditional Norwood procedure. As previously shown, both center and surgeon volume influence outcomes following the Norwood procedure (77); it remains unclear whether hybrid mortality is influenced by institutional HLHS volume (62); thus, low-volume centers may benefit from the use of hybrid procedures as an interim management option when transfer to a higher volume center is not available or would be delayed.

## Superior Cavopulmonary Connection

The superior cavopulmonary connection (SCPC, bidirectional Glenn or hemi-Fontan) has a significant salutary effect on cardiac function. The second-stage procedure relieves the volume load on the single ventricle while maintaining systemic oxygen delivery (78). This results in ventricular work-load approximating that of the systemic ventricle in biventricular circulation (79, 80). One of the critical features of the second-stage procedure is the opportunity for the ventricle to have enough time for ventricular remodeling following the removal of the volume load prior to Fontan completion (24). In addition, the use of an intermediate SCPC provides the opportunity to address other anatomic and physiologic abnormalities (including atrioventricular valve regurgitation, pulmonary artery distortion) therefore optimizing the chances of a successful Fontan completion. Overall, the advantages of performing a second-stage palliation are felt to outweigh its disadvantages, and most centers perform a three-stage palliation for the univentricular heart rather than proceeding directly to Fontan completion.

In light of these physiologic advantages, and aiming to reduce the inter-stage mortality among patients with a systemic-to-pulmonary artery shunt, the timing of the SCPC has been shifted toward an earlier age than the usual 5–6 years (81–83). The high PVR of the early neonatal period clearly precludes the use of a cavopulmonary connection in early life and when performed in the first 2 months of age is likely to result in elevated cavopulmonary pressures and cyanosis (24). Notably, the SCPC has been performed in some special circumstances as early as 8–10 weeks of age with reasonable outcomes but significantly increased resource utilization (84).

Alternatively, patients who have a reliable and controlled source of pulmonary blood flow have the innate advantage of having a pulmonary vascular bed that is protected from high pressure and flow, while affording appropriate oxygen saturations. In this case, the option of a primary SCPC should be considered after 10–12 weeks of age (24).

### Choice of superior cavopulmonary connection: Hemi-fontan or bidirectional glenn

The type of superior cavopulmonary connection is of little importance, as it does not appear to influence long-term outcomes (85). While the hemi-Fontan operation facilitates a subsequent lateral-tunnel Fontan, this procedure is technically more complex. However, it minimizes the dissection and therefore theoretically may reduce the risk of bleeding, lymphatic disruption, or phrenic nerve injury, but no definitive data exist to support these theoretical advantages (86, 87). Although immediate post-operative sinus node dysfunction appears to be more common with the hemi-Fontan, this is a transient phenomenon and subsequent outcomes are equivalent (85). Therefore, the decision regarding which superior cavopulmonary connection to perform should be based primarily on the planned Fontan procedure.

### Additional pulmonary blood flow at second-stage palliation

Controversy continues to exist regarding the management of additional pulmonary blood flow at the time of SCPC. Additional pulmonary blood flow may be provided by either leaving a previous systemic–pulmonary shunt intact at the time of SCPC, or by leaving antegrade flow through a banded or stenotic native pulmonary artery (88, 89). Theoretically, the potential benefits of maintenance of additional pulmonary blood flow include: (1) higher oxygen saturation levels between superior and total cavopulmonary connection procedures (88–94) and (2) improved pulmonary arterial growth (88, 91, 94, 95), and (3) lowering of PVR due to the salutary effects of pulsatile blood flow (96, 97). In addition, as a result of pulsatile flow and higher oxygen levels, there may be a decreased stimulus for the development of pulmonary arteriovenous malformations and systemic-to-pulmonary collateralization (96).

However, there is no convincing evidence to support these benefits. Because of both the increase in venovenous collaterals and somatic growth, the higher oxygen saturations may not persist through Fontan palliation (95, 98, 99). There is no conclusive data to validate the notion of increased growth and development of the pulmonary arterial vasculature (92, 100), or normalization of pulmonary arterial size (88, 101).

Furthermore, preservation of additional pulmonary blood flow has the potential for significant side effects, predominantly related to the persistent volume load on the single ventricle (81). This is especially true of cases in which the additional pulmonary blood flow occurs through a shunt. The ongoing volume load may result in either worsening of atrioventricular valve regurgitation or ventricular function; although, conflicting data exist and some reports have demonstrated the maintenance of both valvular and ventricular function following SCPC with preservation of native antegrade flow through the pulmonary artery (88, 91, 94, 98). In addition, the higher pressure in the superior cavopulmonary connection (89, 91, 93–95) has been associated with a higher incidence of prolonged pleural drainage (89, 93, 94, 102, 103), increased length-of-stay (102), and possibly an increase in the incidence of superior vena caval syndrome (88). In the long-run, these higher pressures also lead to increased venovenous collaterals (94, 95). Finally, volume-loading remains a concern for Fontan candidacy and long-term cardiac function, especially since older age at the time of volume unloading is associated with decreased exercise performance in adolescence (104).

It does not appear that either strategy increases the likelihood of Fontan candidacy (89, 94, 99). Patients with additional pulmonary blood flow may tolerate a longer delay until Fontan completion (99), but the long-term advantages of such a strategy are not clear. Some (89, 102) – though not all (88, 91, 94, 98) – series report higher mortality among patients with additional pulmonary blood flow. It seems clear that, among patients with elevated PVR or hypoxia despite an unobstructed SCPC, preservation of pulmonary blood flow may allow for partial improvement in the volume loading of the ventricle with the possibility of Fontan candidacy in the future. In fact, some have advocated the use of an SCPC with preserved pulmonary blood flow as an alternative to Fontan palliation (103, 105). However, given the restrictive (through either banding or native stenosis) antegrade blood flow, most patients will eventually outgrow the pulmonary blood flow and become cyanotic. Subsequent late Fontan conversion among patients developing worsening cyanosis is associated with high mortality (106), suggesting that it is not the best strategy in patients who are Fontan candidates.

Care should be exercised in situations where the native pulmonary outflow tract is ligated. In these cases, the native pulmonary artery stump has the potential to act as a nidus for thrombus formation (107–109). Various surgical techniques may be used to eliminate the supravalvar area of stasis in the main pulmonary artery. These include the creation of a DKS anastomosis in patients at-risk for later systemic outflow tract obstruction, resection of pulmonary valve leaflets at the time of SCPC, or closure while oversewing the valve (106).

## Total Cavopulmonary Connection – The Contemporary Fontan Procedure

### Physiology of the total cavopulmonary connection

Initially, the Fontan procedure was described for the treatment of tricuspid atresia and was intended to use atrial contractions as a “pulmonary ventricle” (110–112). However, it subsequently became clear that the contractions of the atrium were insufficient (and unnecessary) to provide energy to propulse the blood leaving the systemic capillary beds to perfuse the lungs (113). In the current single-ventricle staging process, the benefits of the Fontan procedure include near normalization of systemic oxygen saturations and elimination of the risk of paradoxical embolization. Unfortunately, this benefit comes at the expense of, chronic passive congestion within the systemic venous system and the liver (114, 115), limited cardiac output reserve both at rest and during exercise (111, 112), and a higher afterload on the ventricle (114).

Nevertheless, in many situations, a TCPC provides the best long-term palliation for patients with a complex cardiac defect not amenable to biventricular repair. Alternatively, it has been suggested that an SCPC (with or without additional antegrade pulmonary blood flow) could be the final palliative stage in patients with unsuitable hemodynamics for Fontan completion or as a means to avoid the long-term ill effects of chronic passive congestion (103, 105). Although currently very little evidence exists to guide the long-term choice of palliation strategy in individual patients, in most centers, the Fontan remains the palliation strategy of choice (24, 115).

### Fontan candidacy and timing

In 1977, Choussat and colleagues published the criteria for an ideal Fontan candidate (Table 2) (116). Since then, these have been refined based on a better understanding of the anatomic and physiologic variables that are indispensable to create a sound and efficient Fontan physiology, and a recognition that certain imperfections can be corrected prior to Fontan completion (110, 117–121). Most importantly, it remains critical that the resistance across the pulmonary capillary bed remains low. Excessive PVR is a clear contraindication to a Fontan because PVR provides the primary limitation to cardiac output in patients without a prepulmonary pump (111, 112), and non-pulsatile flow may result in long-term poorly adaptive remodeling (97, 122). While mean pulmonary artery pressures >15 mm Hg are associated with Fontan failure (117, 118), precise cutoffs have been difficult to identify (in part because few centers will attempt a Fontan in the setting of a Rp >3 or 4) (24, 115). Pulmonary arterial size itself does not appear to present a contraindication to Fontan completion because it is not well correlated with PVR (123), but enlargement of hypoplastic pulmonary arteries should be considered prior to Fontan completion.

**Table 2:** **The “Ten Commandments” of the ideal Fontan candidate**.

Age older than 4 years
Sinus rhythm
Normal systemic venous return
Normal right atrial volume
Mean pulmonary artery pressure <15 mm Hg
Pulmonary arteriolar resistance <4 Wood units/m^2^
Pulmonary artery–aorta ratio more than 0.75
Left ventricular ejection fraction more than 0.60
Competent mitral valve
Absence of pulmonary artery distortion

Both ventricular dysfunction and atrioventricular valve regurgitation also result in a higher risk of Fontan failure (117, 118). In this setting, a lower threshold for intervening on outflow and arch obstructions among Fontan candidates is likely necessary (119). Even mild elevations in afterload and the consequent development of ventricular hypertrophy associated with an increase in end-diastolic pressures may have important long-term negative consequences (124). Thus, early atrioventricular valve repair may be indicated prior to the Fontan procedure (15). Generally, given the physiologic challenges resulting in conversion to a TCPC, optimization of the circulation should be performed during second-stage palliation to minimize the operative insults of the Fontan procedure itself.

### Choice of total cavopulmonary connection: Extracardiac conduit or lateral tunnel

The Fontan procedure has been considerably modified since the description of a direct atriopulmonary connection in a patient with tricuspid atresia by Fontan (3). A variety of atriopulmonary connections were promulgated, but over the long-term significant complications ensued, including right atrial dilatation, thrombosis within the Fontan circuit, compression of the right pulmonary veins, atrial dysrhythmias, and obstruction of the atriopulmonary connection (125). Currently, nearly all “Fontan” procedures are performed as TCPC (126). Most are either the lateral tunnel [initially described by deLeval (127)] or the extracardiac conduit [described by Marcheletti (128)]. Unfortunately, as with many aspects of the Fontan palliation, clarity as to the optimal type of TCPC in all patients (or even in specific subsets) remains elusive. Although the lateral-tunnel technique was widely adopted initially, concerns about the development of sinus node dysfunction and the potential development of atrial dysrhythmias associated with the segment of atrial wall exposed to higher systemic venous pressure, led to a gradual shift toward adoption of the extracardiac conduit (121, 129).

Approximately, 2/3 of all TCPC procedures performed in the United States are extracardiac conduits and the remainder are predominantly lateral tunnels (121). The procedures are different, but it remains largely a matter of speculation as to whether and how the theoretical benefits of each translate into alterations in long-term outcomes. A recent report from the STS Congenital Heart Surgery database identified the use of the extracardiac conduit as a risk factor for early mortality (121). Given the retrospective, observational, large dataset used, it is difficult to know whether this reflects differences in the procedure itself or differences in unmeasured covariates influencing the choice of procedure at many centers.

Among the advantages of the lateral tunnel are the potential for pathway growth enabling early Fontan completion, often between 12 and 30 months of age (121), as well as both theoretical (130, 131) and observed (132, 133) minimization of power loss through the Fontan circuit. In theory, decreasing the power loss through the circuit may improve the functional and exercise capacity in lateral tunnel patients (133), although direct comparisons have shown an advantage to the extracardiac conduit contrary to these predictions (134). Differences between theory and practice may be related to deviations over time and with growth from the ideal tubular structure (138). While earlier Fontan completion may have benefits in reducing the stimulus for aortopulmonary collateral formation by normalizing oxygen saturations, studies have not demonstrated a resultant long-term advantage in survival or functional status (135).

Alternatively, the extracardiac conduit TCPC must be performed at an older age due to the lack of growth potential in the conduit. Some perceived advantages to the procedure include: no need for myocardial ischemia or even cardiopulmonary bypass (135), and smaller suture lines and a reduction in foreign material within the right atrium (135–139). Additionally, multiple variations of the typical procedure have been advocated, including the use of a pericardial tube, “Y”-graft modifications, and a combined intra/extracardiac conduit, among others (140–144).

Overall, the variability in diagnosis, operative technique, and perioperative management as well as limited long-term follow-up continues to plague studies attempting to identify a significant advantage of one technique or another. Whether hybrid approaches to Fontan completion (generally requiring a hemiFontan as the second stage procedure) will ultimately result in better early and late outcomes remains unclear (140, 141).

### Use of a fenestration

In the current era, the majority of Fontan procedures are performed with “fenestration” (121, 129). There is evidence including a prospective randomized trial (145) that fenestration decreases the incidence of prolonged post-operative effusions (145–147), reduce post-operative lengths of hospital stay (146, 148, 149), and lessen the need for early reinterventions (145, 146); however, conflicting views are supported by more recent but non-controlled studies (117, 150–156). The impact of fenestrations on survival and Fontan take-down is less clear (135, 155, 157–163). Early studies appeared to suggest a lower mortality among fenestrated Fontan patients (146, 147, 157, 164), but more contemporaneous series have not identified a higher risk of early mortality associated with the lack of a fenestration (117, 155, 165, 166).

Most centers performing lateral-tunnel Fontans routinely perform fenestrations (155, 158), some do not (159). Technical modifications of the Fontan procedure, including the avoidance of cardiopulmonary bypass and myocardial ischemia, have been pursued in a few centers with attempting to decrease the morbidity after Fontan procedure, but has not been demonstrated (135). While some centers have retreated from the use of a fenestration (166), others are using it with increasing frequency (167). Arguments against routinely leaving incomplete atrial partitioning include: ongoing hypoxemia with a potential continued impetus for collateral formation (168), risk for paradoxical embolus (169), decreased exercise tolerance (170), and the potential for conduit thrombosis following interventional fenestration closure (171, 172). However, many of these risks are only theoretical, and no studies have demonstrated a statistically significant increase in the most important complications including stroke and conduit thrombosis (148, 167).

Closing of fenestrations has not resulted in improvement in exercise capacity (173), and there is no data demonstrating a decreased risk for thromboembolic complications in the absence of a fenestration. Despite objective evidence that support the use of a fenestration to decrease the incidence and duration of pleural effusions, relative agreement about its use only exists in patients considered at high-risk for post-operative morbidity (24). This includes patients with elevated Fontan conduit pressures, extensive systemic-to-pulmonary collateral flow, decreased ventricular function, and elevated PVR (174, 175).

## Long-Term Complications of Univentricular Physiology

The long-term complications of circulatory physiology lacking a pulmonary pump are manifested. As noted above, transition to the TCPC from atriopulmonary connections has ameliorated some of the complications associated with Fontan’s original technique, but has not eliminated them (127, 128). Even with a well-functioning circuit and low PVR, patients with a TCPC have decreased exercise tolerance as a result of limited ability to increase cardiac output, due to the dependency of cardiac output on pulmonary resistance rather than loading or inotropy (111, 112). In addition, cardiac rhythm disturbances are common (176–179), and loss of sinus rhythm may have important deleterious hemodynamic effects (184). Protein-losing enteropathy and plastic bronchitis are thought to be related to chronic exposure to elevated central venous pressures, low cardiac output, and lymphatic congestion (180–182). They constitute two of the most important and debilitating chronic sequelae of the Fontan physiology and are commonly associated with significant mortality (124). It has become increasingly obvious that chronic elevation of central venous pressures is associated with variable levels of hepatic congestion, fibrosis, and even cirrhosis (180). Finally, thromboembolic complications, while more common with atriopulmonary connections, remain an important long-term source of morbidity and mortality in patients with TCPC (124, 183). Thromboembolic disease is likely multifactorial and related to circulatory stasis and alterations in the coagulation system (184). Optimal anticoagulation in these patients remains unclear, although recent retrospective analyses have found reduced morbidity and mortality among patients receiving antiplatelet or warfarin therapy (124). Options include both antiplatelet therapy and anticoagulation with either warfarin or heparin; no specific therapy provides clear benefit in all patients (183).

## Transplantation for Patients with Univentricular Heart

Due to limitation in donor availability, cardiac transplantation cannot provide a comprehensive treatment option for those patients with single-ventricle physiology, but has become the bail-out strategy for selected patients with a failed palliative strategy either with or without preserved ventricular function. Nevertheless, it should be understood that this does not constitute a cure but rather a more manageable form of palliation, which may be associated with a better quality of life (185). In the early era of hypoplastic left heart syndrome treatment, Loma Linda pioneered and popularized the use of transplantation as the primary treatment for these patients (186–188). Outcomes were excellent, and outcomes following transplantation among infants with univentricular heart remain excellent (189, 190). However, these outcomes rely on children reaching transplantation quickly enough to avoid waitlist mortality and to avoid clinical decline or the need for palliative procedures that would alter post-transplant mortality. Given the relative paucity of donor allografts available, transplantation of all patients with univentricular heart is not practical.

Alternatively, transplantation can improve outcomes by eliminating the risk factors, which commonly lead to high mortality among patients with palliated univentricular circulation. For example, severe atrioventricular valve regurgitation or ventricular dysfunction significantly increases the risk of single-ventricle palliation (15), but unless PVR was affected (191), these would have little impact on outcomes following orthotopic heart transplantation. In contrast, low birth weight and early gestational age are risk factors for palliation that would not be eliminated by transplantation and would influence post-transplant outcomes (67, 192, 193). Similar consideration may be given to patients with right ventricular-dependent coronary circulation pulmonary atresia with intact ventricular septum. Within the confines of the current availability of donor allografts for children, a selective strategy of transplantation in these patients is likely to offer the best chance at long-term survival in all patients.

Even the “perfect” Fontan has a recognized attrition rate over time (194). The exact definition of Fontan failure is imprecise, but should include both functional and hemodynamic evaluation (195). Transplantation provides an important option for Fontan failure, but remains a high-risk procedure, especially in adults (196–200). Precise criteria for listing and the optimal timing of transplantation in patients with declining functional status remain unclear (198, 201). Patients with anatomic issues that lead to energy loss or rhythm disturbances may benefit from Fontan revision or conversion, or atrial arrhythmia surgery (178, 195). While patients with impaired ventricular function appear to benefit from transplantation, patients with Fontan failure in the setting of preserved ventricular function have poor outcomes independent of transplantation (201). Whether this represents inappropriate timing of transplantation (where earlier transplant would have resulted in better outcomes) or the fact that ongoing alterations in pulmonary vascular and hepatic function in particular are not improved with pump replacement remains unclear. The need for simultaneous liver transplantation is also an area of active investigation with no clear threshold for when hepatic dysfunction should be considered irrecoverable. Mechanical circulatory support in Fontan patients also remains challenging and the optimal strategy of support to patients with a TCPC will likely depend on the type of failure (circuit failure versus ventricular pump failure) (201–203). The increasing number of adults with imperfect Fontan circulations is leading to active investigation to answer these questions in order to improve the survival and functional outcomes in these patients with or without transplantation.

## Univentricular vs. Biventricular Circulation

Despite the binary nature of the surgical decision regarding univentricular palliation, “univentricular” lesions represent a spectrum of anatomic subtypes, which – at the milder end – may be amenable to a two-ventricle repair. The definition of “milder end” and the determination of whether a borderline anatomy is adequate to pursue a biventricular circulation remain challenging. Different level of obstruction in these borderline cases makes the evaluation difficult and the surgical intervention to address them a challenge. While obstruction at the aortic level and even at the level of the systemic outflow can be effectively solved, it is often the inflow in the ventricular cavity (size of the mitral or atrioventricular valve) that presents the greatest challenge to determine the feasibility of a two-ventricle repair. However, the decision about which patients may benefit from a two-ventricle repair remains highly dependent on the specific lesion and the institutional expertise to accomplish these complex repairs with acceptable morbidity and mortality.

### HLHS

Patients with left heart anomalies represent a wide spectrum of anatomy and physiology. The decision-making is fairly clear at ends of the spectrum, but becomes more difficult when patients fall in the so-called borderline category. Despite extensive studies (204–208), there are currently no definitive criteria to identify which patients are likely to benefit from a single or a biventricular strategy. Obstruction to systemic cardiac output may occur at multiple levels within the left-sided circulation: including the mitral valve, the left ventricular chamber (through both underdevelopment and presence of endocardial fibroelastosis), the left ventricular outflow tract, the aortic valve, and the aorta itself. Each level needs to be examined to determine whether reconstruction is necessary and if so, whether the obstruction can be relieved. While data from the CHSS^1^ may provide guidance to determine the optimal strategy based on preoperative criteria including aortic diameter, tricuspid regurgitation, LV size, and the presence of EFE, there remains significant cross-over between the predicted best management and actual management (206–208).

In most cases, aortic atresia, mitral atresia, and extremely small left ventricles (left ventricular end-diastolic volume *z*-score <−5) are clear indications for single-ventricle palliation. While some centers have had success with staged left ventricular recruitment in patients with left-sided structures with *z*-scores as low as −5, this is a technically challenging process requiring multiple procedures and the long-term results compared to single-ventricle palliation are uncertain (209). Based on CHSS data, the need for early re-intervention in patients selected for a biventricular strategy predicts poor outcomes, suggesting that in borderline cases, univentricular palliation may be the safer strategy – at least in the early to mid-term follow-up (207).

When left ventricular outflow obstruction is present, multiple procedures to enlarge the systemic outflow and/or to address the aortic valve can be performed, including surgical or balloon aortic valvuloplasty, the Ross–Konno procedure (174, 210–212), and the Yasui operation (212, 213). Similarly, resection of endocardial fibroelastosis can have a favorable effect not only in ventricular compliance and function but also in growth (209, 214, 215). In the absence of precise definitions of an adequate LV outflow, a multitude of criteria are used to predict the adequacy of the left heart to support the systemic circulation. These include an LV outflow tract dimension greater than the patient’s weight in kilograms (216), aortic annular diameter >4.5 mm, or *z*-score >−5 (205), It is the mitral (or left-sided AV valve in the case of unbalanced AV canal) that constitutes the greatest challenge when deciding toward a two-ventricle reconstruction in borderline cases. In this scenario, the unanticipated need for a mitral valve repair/replacement following a Ross–Konno procedure can be associated with a significant increase in mortality (174, 175).

### Pulmonary atresia/intact ventricular septum

In patients with pulmonary atresia and intact ventricular septum, it is the size of the tricuspid valve that provides the best guidance toward an appropriate management pathway. Outcomes following a biventricular strategy (transannular patch and systemic–pulmonary artery shunt) are significantly worse when the tricuspid valve *z*-score is below −4, supporting the notion that the tricuspid valve size provides the most reliable indicator of the adequacy of the right heart for biventricular repair (217–220). However, the deleterious effect of aggressively pursuing a biventricular strategy can extend beyond the early follow up, as mid-term functional outcomes are worse when patients with a small tricuspid valve *z*-score are forced down to a biventricular management pathway (221). When it is evident that the right heart cannot manage the entire cardiac output, significant enthusiasm exists about the possibility of maintaining antegrade pulmonary blood flow form the inferior vena cava while avoiding the exposure of the hepatic circulation to the higher venous pressures of the Fontan circulation in a so-called 11/2 ventricle repair (superior cavopulmonary connection while leaving antegrade pulmonary flow through the pulmonary valve). Although it has been postulated that this physiology would be associated with an improved functional status, follow-up studies suggest that these patients have exercise capacities and cardiac reserves similar to univentricular patients (222). Patients with larger tricuspid valve (*z*-score >−2.5), a tripartite right ventricle, and a patent pulmonary valve can undergo an initial biventricular repair with excellent results although a systemic–pulmonary shunt is not infrequently needed to provide adequate pulmonary blood flow in the neonatal period (217).

A particularly challenging group is the one comprised by patients with PA/IVS and right ventricular dependent coronary circulation, in whom single-ventricle palliation carries a very high risk, not only initially but during subsequent palliation (217, 223). This is especially true in the presence of a vessel decompressing the coronary circulation into the pulmonary artery and during the construction of a systemic-to-pulmonary artery shunt (223). Although there is no clear data demonstrating which management pathway is best in these challenging patients, primary transplantation appears to be the preferred option in many centers (224).

### Unbalanced atrioventricular septal defect

Surgical decision-making regarding the pursuit of a biventricular circulation in patients with unbalanced atrioventricular septal defects is particularly difficult and depends on the adequacy of the AV valve inflow. Detailed analysis of echocardiographic variables aimed to identify which patients would have a successful biventricular repair has been facilitated by the creation of the modified atrioventricular valve index (AVVI, an echocardiographic measure of the relative area of the left atrioventricular valve in relation to the entire AV valve) (225). While a modified AVVI between 0.4 and 0.6 identified the balanced range of the spectrum, patients with an AVVI between 0.2 and 0.4 (slightly unbalanced with right-dominance) exhibited heterogeneity of management strategies associated with a cluster of poor outcomes, which illustrates the difficulty of this decision (225). Additional analysis has identified that the inflow angle between the right and left AV valves and the septum [right ventricle/left ventricle inflow angle (226)] may be important in guiding early operative decision-making. However, there is a paucity of data validating any of these individual measures.

### Making the decision for univentricular or biventricular reconstruction

While case series may provide evidence of what can be done by a specific surgical team, they do not necessarily provide data to support a particular pathway at all centers. In this context, multi-institutional studies, such as those from the CHSS (206, 207, 221, 225, 226), may provide the most broadly applicable data. Ultimately, the decision to pursue a univentricular or biventricular strategy must be based on the experience of the individual surgeon and center.

## Summary

Despite the advances in the operative and perioperative management of patients with univentricular heart, morbidity and mortality remain high. Decisions made in the early post-natal period may have important consequences for both early survival and long-term morbidity. In particular, the surgical strategy chosen – whether univentricular palliation or biventricular repair, hybrid versus traditional procedures, choices regarding the most appropriate pulmonary blood flow – will affect management throughout a patient’s life. Unfortunately, there is lack of conclusive data to guide these choices. Further studies are required in many areas to identify determinants of the optimal surgical strategy in individual patients. In the absence of definitive data, surgical decisions must rest on subjective assessment grounded in a thorough understanding of the anatomy, physiology, and potential consequences of each strategy.

## Conflict of Interest Statement

The authors declare that the research was conducted in the absence of any commercial or financial relationships that could be construed as a potential conflict of interest.

## Footnotes

^1^Online calculator available at: http://www.chssdc.org/content/chss-score-neonatal-critical-aortic-stenosis
